# Supplementary material for: Radiomics-Based Deep Learning Prediction of Overall Survival in Non-Small-Cell Lung Cancer Using Contrast-Enhanced Computed Tomography
Source: Cancers (Basel). 2022 Aug 4;14(15):3798. doi: 10.3390/cancers14153798 (PMC9367244; doi:10.3390/cancers14153798)
Supplement: Supplementary file 1 [file cancers-14-03798-s001.zip › cancers-1823011-supplementary.pdf]

Supplementary Materials

Radiomics-Based Deep Learning Prediction of Overall Survival in Non-Small Cell Lung Cancer Using Contrast-Enhanced Computed Tomography

Supplementary Table S1. Demographics of patients' clinical data (full dataset)

Characteristics	CGH (n=338)	SCGH (n=154)	P
**Age(y)**			0.56
Mean ± SD	66.98 ± 12.24	66.62 ± 11.84	
**Gender, no. (%)**			0.75
Male	183 (54.14%)	81 (52.60%)	
Female	155 (45.86%)	73 (47.40%)	
**Histology**			0.16
Adenocarcinoma	218 (64.50%)	111 (72.08%)	
Squamous cell carcinoma	73 (21.60%)	23 (14.94%)	
Adenosquamous carcinoma	11 (3.25%)	1 (0.65%)	
Large cell cancer	4 (1.18%)	2 (1.30%)	
Other NSCLC	32 (9.47%)	17 (11.04%)	
**Laterality**			0.28
Right	184 (54.44%)	89 (57.79%)	
Left	149 (44.08%)	65 (42.21%)	
Both side lesion, unknown origin	5 (1.48%)	0 (0.00%)	
**Primary Site**			0.38
Upper lobe	188 (55.62%)	93 (60.39%)	
Middle lobe	23 (6.80%)	11 (7.14%)	
Lower lobe	109 (32.25%)	46 (29.87%)	
Lung	15 (4.44%)	2 (1.30%)	
Overlapping	3 (0.89%)	2 (1.30%)	
**Clinical T stage**			0.47
0	1 (0.30%)	0 (0.00%)	
1	40 (11.83%)	18 (11.69%)	
2	93 (27.51%)	32 (20.78%)	
3	70 (20.71%)	39 (25.32%)	
4	134 (39.64%)	65 (42.21%)	
**Clinical N stage**			0.59
0	108 (31.95%)	54 (35.06%)	
1	27 (7.99%)	11 (7.14%)	
2	85 (24.85%)	44 (28.57%)	
3	118 (34.91%)	45 (29.22%)	
**Clinical M stage**			0.07
0	110 (32.54%)	63 (40.91%)	
1	228 (67.46%)	91 (59.09%)	
**Clinical stage**			0.16
I	48 (14.20%)	23 (14.94%)	
II	12 (3.55%)	11 (7.14%)	

III	50 (14.79%)	29 (18.83%)	
IV	228 (67.46%)	91 (59.09%)	
**Pathological T stage**			0.68
0	3 (0.89%)	2 (1.30%)	
1	30 (8.88%)	13 (8.44%)	
2	42 (12.43%)	17 (11.04%)	
3	9 (2.66%)	6 (3.90%)	
4	3 (0.89%)	4 (2.60%)	
Not available	251 (74.26%)	112 (72.73%)	
**Pathological N stage**			0.01*
0	54 (15.98%)	23 (14.94%)	
1	3 (0.89%)	6 (3.90%)	
2	20 (5.92%)	7 (4.55%)	
3	16 (4.73%)	0 (0.00%)	
Not available	245 (72.49%)	118 (76.62%)	
**Pathological M stage**			0.01*
0	0 (0.00%)	0 (0.00%)	
1	63 (18.64%)	15 (9.74%)	
Not available	275 (81.36%)	139 (90.26%)	
**Pathological stage**			1.52E-05*
1	42 (12.43%)	22 (14.29%)	
2	9 (2.66%)	9 (5.84%)	
3	23 (6.80%)	30 (19.48%)	
4	81 (23.96%)	91 (59.09%)	
Not available	183 (54.14%)	2 (1.30%)	
**Surgery**			0.66
None	250 (73.96%)	111 (72.08%)	
Yes	88 (26.04%)	43 (27.92%)	
**Chemotherapy**			0.06
None	202 (59.76%)	78 (50.65%)	
Yes	136 (40.24%)	76 (49.35%)	
**Radiation therapy**			0.02*
None	217 (64.20%)	115 (74.68%)	
Yes	121 (35.80%)	39 (25.32%)	
**Systemic therapy**			0.08
None	112 (33.14%)	39 (25.32%)	
Yes	226 (66.86%)	115 (74.68%)	
**Targeted therapy**			0.36
None	215 (63.61%)	104 (67.53%)	
Yes	123 (36.39%)	50 (32.47%)	
**Smoke**			0.91
None	188 (55.62%)	88 (57.14%)	
Yes	138 (40.83%)	66 (42.86%)	
Not available	12 (3.55%)	0 (0.00%)	
**Betel nut use**			5.25E-05*
None	300 (88.76%)	114 (74%)	
Yes	27 (7.99%)	22 (14.3%)	
Not available	11 (3.25%)	18 (11.7%)	
**Alcohol use**			4.00E-03*
None	240 (71.01%)	92 (59.74%)	
Quit drinking	33 (9.76%)	18 (11.69%)	
Sometimes	20 (5.92%)	11 (7.14%)	
Always	34 (10.06%)	15 (9.74%)	

Not available	11 (3.25%)	18 (11.69%)	
**Visceral Pleural Invasion**			5.43E-15*
None	310 (91.72%)	97 (62.99%)	
Pleural invasion	28 (8.28%)	57 (37.01%)	
**Assessment of performance status before treatment**			9.73E-15*
ECOG=0；KPS=100。	73 (21.60%)	29 (18.83%)	
ECOG=1；KPS=80 / 90。	9 (2.66%)	38 (24.68%)	
ECOG=2；KPS=60 / 70。	14 (4.14%)	11 (7.14%)	
ECOG=3；KPS=40 / 50。	5 (1.48%)	3 (1.95%)	
ECOG=4；KPS=10 / 20 / 30	1 (0.29%)	5 (3.25%)	
Not available	236 (69.82%)	68 (44.16%)	
**Malignant Pleural Effusion**			0.15
None	121 (35.80%)	60 (38.96%)	
MPE diagnosed by physicians and images, without cytology	91 (26.92%)	29 (18.83%)	
Not available	126 (37.28%)	65 (42.21%)	
**Survival**			0.20
Alive	97 (28.70%)	53 (34.42%)	
Dead	241 (71.30%)	101 (65.58%)	
**Duration (months)**	20.15 ± 19.95	12.75 ± 11.70	2.22E-05*
Median	12	11	

Note: p-values are results of $\chi 2$ test for categorized variables and student's t-test for continuous variables.

NSCLC= non-small cell lung cancer, ECOG=Eastern cooperative oncology group, KPS= Karnofsky scale. *P < 0.05

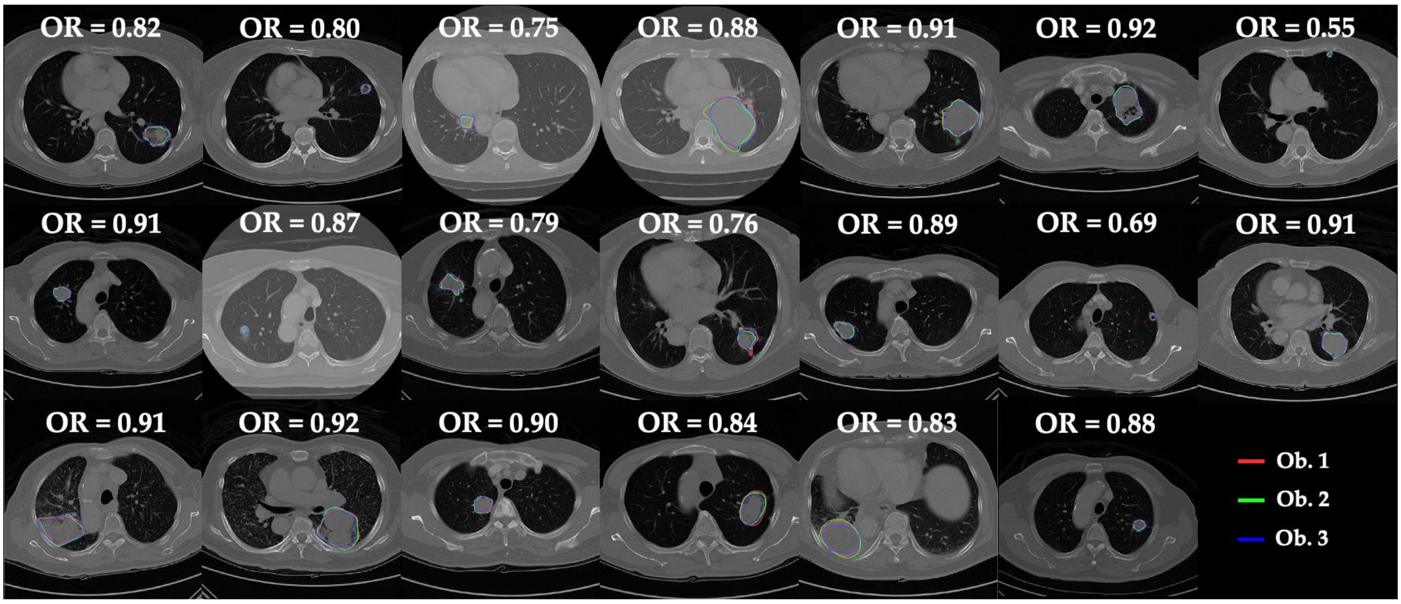

Supplementary Figure S1. Overlapping rate of tumor segmentation among the three observers. To assess the inter-observer variation, 20 NSCLC tumors were randomly selected and the ROIs of these tumors were delineated by the three radiologists. The red, green and blue contours indicate manual segmented tumors by three observers respectively. The ICC of overlapping rate is 0.83±0.10 which shows high consistency. OR: overlapping rate, Ob.: observer.

Supplementary Table S2. Intraclass correlation coefficient (ICC) of 19 radiomic feature categories. ICC of radiomic features were also assessed to verify feature reproducibility. Texture features with HLL wavelet filter performed lower consistency among three observers.

**Wavelet**	**Categories**	**ICC**	
		Mean	Standard deviation
Primary	Histogram	0.897	0.085
Primary	Geometry	0.885	0.104
Primary	Texture	0.855	0.125
LLL	Histogram	0.916	0.062
LLL	Texture	0.889	0.089
LLH	Histogram	0.935	0.143
LLH	Texture	0.893	0.122
LHL	Histogram	0.917	0.095
LHL	Texture	0.869	0.132
LHH	Histogram	0.940	0.085
LHH	Texture	0.901	0.105
HLL	Histogram	0.940	0.107
HLL	Texture	0.819	0.244
HLH	Histogram	0.966	0.069
HLH	Texture	0.868	0.161
HHL	Histogram	0.881	0.179
HHL	Texture	0.868	0.146
HHH	Histogram	0.934	0.105
HHH	Texture	0.897	0.101

Supplementary Table S3. Comparisons between patients with and without targeted therapy.

Characteristics	Non-Targeted therapy (n=319)	Targeted therapy (n=173)	P values
**Histology**			1.79×10^{-14}
Adenocarcinoma	174	155	
Squamous cell carcinoma	93	3	
Adenosquamous carcinoma	10	2	
Large cell cancer	6	0	
Other NSCLC	36	13	
**Clinical T stage**			2.34×10^{-04}
0	1	0	
1	50	8	
2	89	36	
3	59	50	
4	120	79	
**Clinical N stage**			2.18×10^{-03}
0	124	38	
1	23	15	
2	75	54	
3	97	66	
**Clinical stage**			2.29×10^{-20}
I	71	0	
II	23	0	
III	66	13	
IV	159	160	
**Surgery**			3.43×10^{-13}
None	200	161	
Yes	119	12	
**EGFR mutation**			1.76×10^{-49}
Absent	79	32	
Present	25	122	
Not available	215	19	
**Overall survival (months)**	16.93 ± 17.55	19.51 ± 19.01	0.13
